# Regulatory non-coding RNAs: Emerging roles during plant cell reprogramming and *in vitro* regeneration

**DOI:** 10.3389/fpls.2022.1049631

**Published:** 2022-11-10

**Authors:** Daniela Cordeiro, Jorge Canhoto, Sandra Correia

**Affiliations:** Centre for Functional Ecology, Department of Life Sciences, University of Coimbra, Coimbra, Portugal

**Keywords:** embryogenic potential, lncRNAs, miRNAs, plasticity, pluripotency, somatic embryogenesis, totipotency

## Abstract

Plant regeneration is a well-known capacity of plants occurring either *in vivo* or *in vitro*. This potential is the basis for plant micropropagation and genetic transformation as well as a useful system to analyse different aspects of plant development. Recent studies have proven that RNA species with no protein-coding capacity are key regulators of cellular function and essential for cell reprogramming. In this review, the current knowledge on the role of several ncRNAs in plant regeneration processes is summarized, with a focus on cell fate reprogramming. Moreover, the involvement/impact of microRNAs (miRNAs), long non-coding RNAs (lncRNAs) and small-interfering RNAs (siRNAs) in the regulatory networks of cell dedifferentiation, proliferation and differentiation is also analysed. A deeper understanding of plant ncRNAs in somatic cell reprogramming will allow a better modulation of *in vitro* regeneration processes such as organogenesis and somatic embryogenesis.

## Introduction

Plant propagation through *in vitro* culture techniques, usually known as micropropagation, is a useful biotechnological set of tools that can contribute to satisfying the food and aesthetical needs of the growing world population, mainly, for horticultural plants, such as fruits, vegetables and ornamentals. However, most underlying mechanisms remain unclear and achieving an effective *in vitro* regeneration, *via* somatic embryogenesis (SE) or organogenesis, is still challenging for many species. Besides the huge natural variation in plant regeneration capacity between and within species and/or genotypes (Lardon and Geelen, 2020), many obstacles and difficulties prevent reaching suitable protocols to induce plant cell reprogramming and effective regeneration. These bottlenecks may be related to more technical aspects such as contamination or browning of the explants, and/or to different settings requirements (nutrients, plant growth regulators, solidifying agents, pH, osmotic stress, light and temperature) (Abdalla et al., 2022). But they can also be related to the *in vitro* regeneration process itself, for instance, the difficulty of *in vitro* establishment and propagation, the recalcitrance or low explant responsivity, which can depend on the plant donor age and/or type, on the oxidative state of the explant, on callus proliferation without organogenic or embryogenic ability, on the difficulty in maintaining and proliferating embryogenic cultures or on the formation of abnormal somatic embryos (Correia et al., 2012a; Martínez et al., 2019). Such constraints cause *in vitro* regeneration processes to be frequently based on trial-and-error experiments, which reduces their effectiveness. Both organogenesis and SE can be induced directly in the explant or through a two-step process in which a callus phase first occurs followed by shoot or somatic embryo formation from the callus cells (Long et al., 2022). In any of the regeneration pathways, a complex series of events occurs that is controlled by genetic and epigenetic mechanisms that are still poorly understood (Ikeuchi et al., 2019). Moreover, either organogenesis or SE involve cell proliferation and further differentiation with the formation of shoot meristems or embryos that go through different morphological stages until a complete plant could be obtained. For example, in the case of SE, totipotency must be acquired, embryo development must go through different morphological phases (globular, hearth-shaped, torpedo and cotyledonary), and maturation must be achieved before somatic embryos germinate into plantlets (Méndez-Hernández et al., 2019). This complex series of interconnected events must be precisely controlled for the regeneration process to be successful. Among these factors are physiological, cellular, biochemistry and genetic factors. Understanding the molecular framework of these processes is crucial for improving protocols and overcoming those hindrances for several crops. In this review, we focused on a field of investigation that has been assuming an increasing relevance in the understanding of the mechanisms involved in developmental transitions, more specifically the regulatory role of non-coding RNAs (ncRNAs) on cell reprogramming and *in vitro* plant regeneration. This field has undergone important advances in animal cell systems, but in plants it remains little explored.

**Figure 1:**
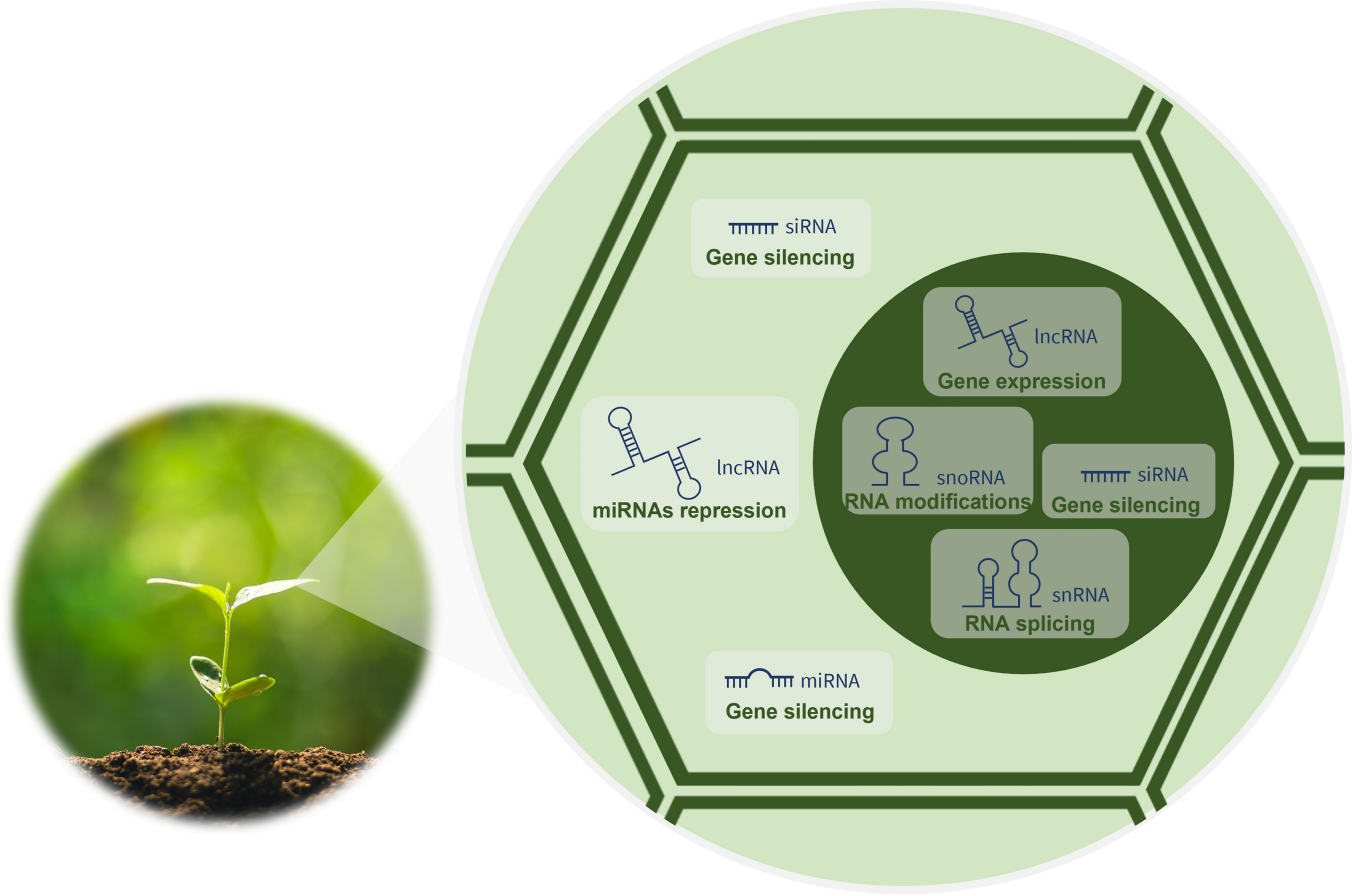
Functions of regulatory ncRNAs in plant cells.

The development of advanced technologies for sequencing and characterizing RNA has revealed key players in the biology of organisms hitherto unknown. Currently, it is known that the eukaryotic cell transcriptome encompasses a wide variety of RNA forms that differ in their biogenesis, mode of action and function. Messenger RNAs (mRNAs) are very well-known RNAs, as they are the templates for protein synthesis. However, these RNAs represent only around 2% of the 90% of the eukaryotic genome that is transcribed into RNA (Rai et al., 2019). The remaining 98% of the transcriptome has no protein-coding capacity and is transcribed from what was previously called junk DNA (Ariel et al., 2015). Nevertheless, it is now known that these genetic information-containing molecules are functional and grouped into two categories (Waititu et al., 2020): the housekeeping and the regulatory ncRNAs. The housekeeping, also called infrastructural or constitutive ncRNAs, are abundant in all cell types and include ribosomal RNAs (rRNAs), transfer RNAs (tRNAs), small nuclear RNAs (snRNAs) and small nucleolar RNAs (snoRNAs). Being constitutively expressed, these ncRNAs regulate generic basic cellular functions maintaining them (Zhang et al., 2019a). In turn, the regulatory ncRNAs modulate gene expression at transcriptional, post-transcriptional and epigenetic levels (Zhang et al., 2019a) as indicated in **Figure 1**. They include transcripts longer than 200 nucleotides (nt), the long ncRNAs (lncRNAs), but also RNAs shorter than 30 nt in length, the small RNAs (sRNAs). In plants, sRNAs, such as microRNAs (miRNAs), small-interfering RNAs (siRNAs) and phased siRNAs (phasiRNAs), have been recently discovered and characterized (Borges and Martienssen, 2015).

The regulatory functions of plant ncRNAs in several biological processes are arousing a growing interest (Böhmdorfer and Wierzbicki, 2015; Yu et al., 2019; Bhogireddy et al., 2021; Song et al., 2021). In turn, in animals, ncRNAs are known as regulators of embryogenesis since they are responsible for developmental transitions and have a pivotal role in cell fate determination, controlling the specification and differentiation of cell types and organ morphogenesis (Pauli et al., 2011; Kanwal et al., 2020). Moreover, ncRNAs are proving to be indispensable in somatic cell reprogramming, specifically in the acquisition and maintenance of pluripotency (Luginbühl et al., 2017).

The ability of some organisms to return some cell types to a meristematic state and regenerate new tissues, organs or whole organisms during post-embryonic development is particularly remarkable in plants (Ikeuchi et al., 2016; Kareem et al., 2016). Their developmental plasticity is used by some species as a reproductive strategy or to survive damage for instance, by the replacement of a lost part after wounding (Perianez-Rodriguez et al., 2014; Ikeuchi et al., 2016). *In vitro*, plant regeneration can be achieved by culturing differentiated tissues or organs in a medium with appropriate nutrients and plant growth regulators. This can occur through three mechanisms: 1) SE, a process in which a somatic cell gives rise to an embryo that later develops into a complete organism (expression of totipotency); 2) organogenesis, which involves the formation of new organs from mature non-meristematic tissues (reflecting pluripotency) and 3) proliferation of axillary shoot meristems (Kumar, 2011). Thus, induced SE and organogenesis processes are noteworthy systems to study the molecular mechanisms underlying plant cell reprogramming, including ncRNA-mediated regulation.

Being sessile organisms, cell reprogramming is required by plants to cope with the multitude of edaphic-climatic stresses they experience during post-embryonic development and for the expression of totipotency and/or pluripotency. In induced regeneration processes, cell reprogramming is also required and can be achieved directly, with no dedifferentiation stage, or indirectly, involving major reprogramming, depending on culture conditions (Paul et al., 2011; Acanda et al., 2013; Sánchez and Dallos, 2018; Zhang et al., 2021). (Atta et al., 2009; Sugimoto et al., 2010; Chandler, 2011; Perianez-Rodriguez et al., 2014). Furthermore, changes in cell fate are accompanied by deep changes in metabolic pathways (Kanwal et al., 2020). In animal cells, for example, a general metabolic switch from oxidative phosphorylation to glycolysis is required for reprogramming and to acquire the ability to proliferate indefinitely (Wu et al., 2016). Likewise, *Araucaria* cell lines with high embryogenic potential were associated with the presence of glycolysis-related proteins (dos Santos et al., 2016). Reinforcing the need for energy metabolism for the achievement of embryogenic potential, glucose metabolism-related proteins were predominantly expressed during the SE of tamarillo (Correia et al., 2012b). Moreover, glycosylases are involved in DNA demethylation (Zhao and Chen, 2014), necessary for cell reprogramming as described later in this review.

Despite being reported as key regulators in plant SE (Chen et al., 2018; López-Ruiz et al., 2019a; Siddiqui et al., 2019; Alves et al., 2021), the ncRNAs’ involvement in plant cell plasticity is still far from being fully understood. Nevertheless, the increasing evidence of the importance of the ncRNAs-mediated regulation in cell pluripotency and regeneration ability brings to light important questions to discuss, such as: (1) To what extent do ncRNAs impact plant regeneration processes? (2) Which regulatory networks are most involved/affected? (3) How can this knowledge be used to modulate induced regeneration processes? Thus, in this review, we highlight the current knowledge on the role of several ncRNAs on the intrinsic plant pluripotency and regeneration capacity, focusing mainly on the regulatory roles of miRNAs, siRNAs and lncRNAs.

## miRNAs

miRNAs are the most abundant class of sRNAs and are encoded by *MIR* genes and transcribed by RNA POLYMERASE II (Pol II). Despite its tiny length of only 20-22 nt, these molecules are involved in gene expression regulation, acting post-transcriptionally by cleaving their target mRNA transcripts or causing translational repression (Yu et al., 2017). miRNAs are involved in almost every intracellular event, despite being highly tissue type-dependent and, therefore, their expression patterns and timings vary among species and throughout development. Although essential for plant meristem maintenance, growth and proliferation control, miRNAs are also emerging as key players in somatic to embryonic cell reprogramming, given their capacity in modulating gene expression (López-Ruiz et al., 2019a).

**Figure 2:**
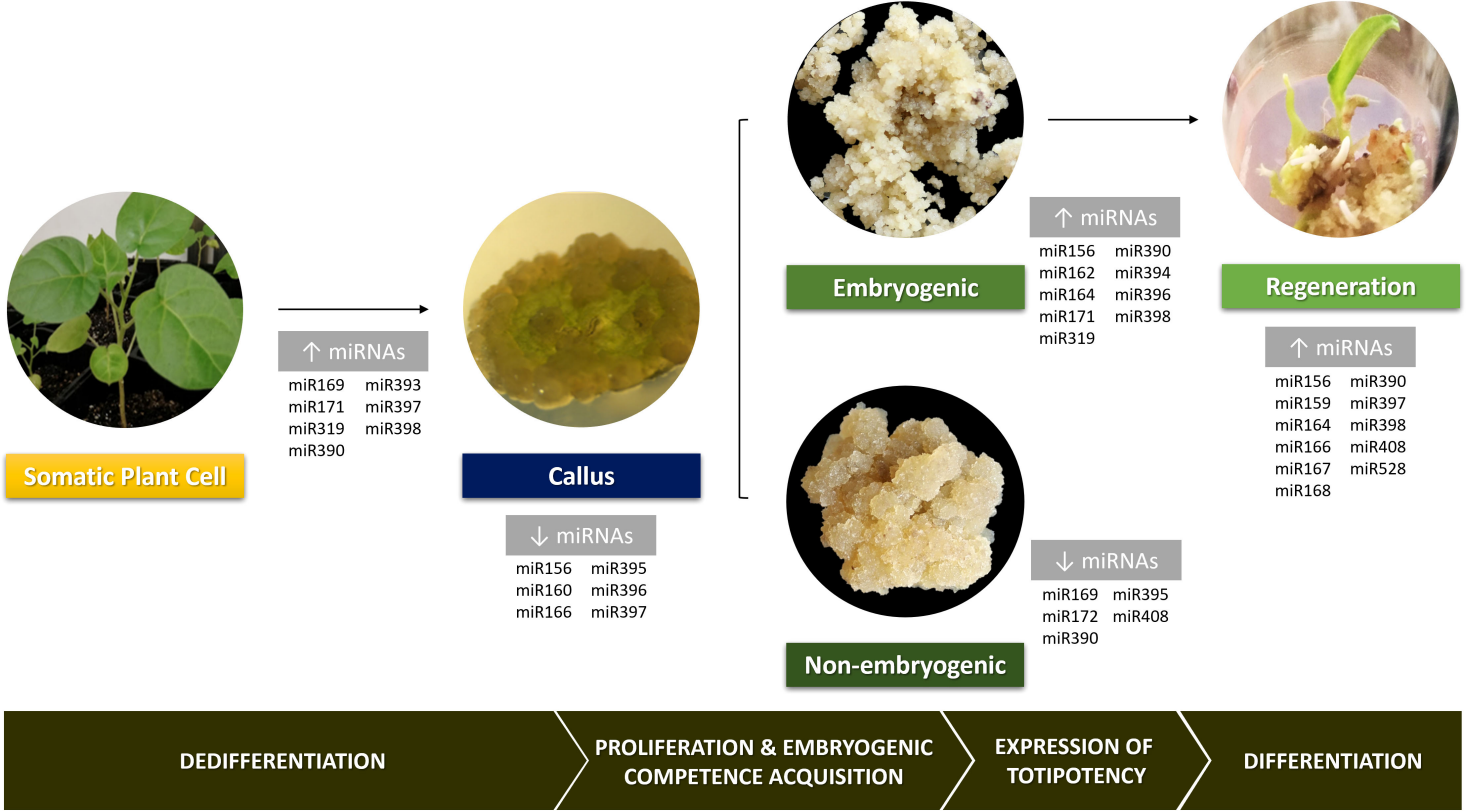
General overview of the involvement of several miRNAs families in various stages of plant cell reprogramming during *in vitro* indirect somatic embryogenesis. Pictures of *Solanum beataceum* illustrate key stages of the regeneration process.

Kanwal et al. (2020) showed that variation in miRNAs expression can enhance or inhibit the reprogramming towards diverse cell types. Also, numerous studies in a wide range of plant species have pointed out differential miRNAs expression in tissues undergoing not only dedifferentiation but also further regeneration (**Figure 2**). During the dedifferentiation phase, plant tissues present a general increase in miRNAs levels (Luo et al., 2006; Szyrajew et al., 2017). Nevertheless, when the callus phase is reached, some miRNAs exhibit low expression levels, as is the case of miR156, miR160, miR166, miR395, miR396 and miR397, which might be a response to the dedifferentiation stimulus (Gao et al., 2019; Juárez-González et al., 2019). Tissues with high embryogenic potential show high miRNAs levels compared to less embryogenic ones, mainly miR156, miR164 and miR394 in maize (López-Ruiz et al., 2019b). This suggests that these miRNAs are likely enhancers of the embryogenic response and modulation of their expression in non-embryogenic tissues could overcome recalcitrance. Besides the low miRNAs levels in non-embryogenic callus, some of them were reported as up-regulated in this type of calluses such as miR169, miR172, miR390, miR395 and miR408 (Zhang et al., 2010; Wu et al., 2015). Silencing these miRNAs in this kind of calluses or tissues could enhance embryogenic competency and ultimately its expression. When hormone depletion occurs in plant regeneration, there is a slight decrease in the miRNAs amount followed by a new accumulation (López-Ruiz et al., 2019b). Surprisingly, tissues in either the callus or plant regeneration phase show a high accumulation of miRNA targets, similarly to what happens with miRNA levels (López-Ruiz et al., 2019b).

Recently, in explants going through later developmental stages and, hence, with lower embryogenic potential, it was found a decrease in the levels of development-related miRNAs and an accumulation of stress- and nutrient- transport-related miRNAs (Juárez-González et al., 2019). This suggests that these last miRNAs are blocking embryogenic potential acquisition, preventing totipotency achievement, probably due to imposed stress and hormone concentration during dedifferentiation. Indeed, unlike moderate stress, which is beneficial for dedifferentiation, excessive stress causes loss of totipotence (Peng et al., 2020).

Cell dedifferentiation goes along with cell wall loosening, which might be associated with miR397-mediated down-regulation of laccases, enzymes involved in cell wall lignification and thickness (Constabel et al., 2000; Sun et al., 2018). Indeed, the levels of expression of miR397 increase during the initial phases of the SE process (López-Ruiz et al., 2019a). In callus tissue, miR397 expression undergoes a slight decline allowing accumulation of laccases and hence lignin deposition, which can be explained by the fact that this tissue contains lignified parenchyma cells (Cao et al., 2020). During plant regeneration phases, miR397 accumulates continually, reaching the highest levels in the adventitious shoots, suggesting a major regulatory role in morphogenesis during advanced differentiation (Liu et al., 2014). Curiously, in conifer, miR397 predominate in somatic embryos but greatly decreased in zygotic ones (Rodrigues et al., 2019).

As miR397, miR160 and miR166 were also defined as key repressors of callus initiation, by modulating the interplay between auxin and cytokinin during organogenesis and through regulation of auxin biosynthesis and response genes, respectively (Cao et al., 2020). Nevertheless, miR160 was proven to be engaged in the embryogenic transition targeting AUXIN RESPONSE FACTORS (ARF) 10 and 16 and contributing to the LEAFY COTYLEDON2-mediated auxin-related pathway induced in *Arabidopsis thaliana* SE (Wójcik et al., 2017). Also contributing to this pathway is the miR65/166 that targets PHABULOSA/PHAVOLUTA and is involved in meristem maintenance (Wójcik et al., 2017). Despite the decrease in the expression levels of these three miRNAs (miR160, miR166 and miR397) during the change from differentiated cells to callus stages, they increase their expression at further differentiation stages (López-Ruiz et al., 2019b).

miR156, highly conserved in plants and targeting SQUAMOSA PROMOTER BINDING PROTEIN-LIKE genes, was confirmed to be involved in callus formation by regulating the cytokinin signalling pathway in citrus (Long et al., 2018; Cao et al., 2020), in modulating redifferentiation during induction of organogenesis in *Acacia crassicarpa* (Liu et al., 2014), in *in vivo* regeneration in tomato (Cao et al., 2020), and also in the ability to form somatic embryos from citrus embryogenic callus (Chen and Zhang, 2016).

Accumulation of miR159a, miR319a, miR162, miR171q, miR390a and miR396 seems to be required for embryogenic callus formation in *A. crassicarpa* organogenesis (Liu et al., 2014) and rice SE (Luo et al., 2006). miR159, which was reported as the most abundantly expressed in tissues under regeneration (Cao et al., 2020), also modulates gene expression during embryogenic callus differentiation in several species such as *A. crassicarpa*, larch and longan (Zhang et al., 2012; Lin and Lai, 2013; Liu et al., 2014).

After hormone depletion, miR156, miR164, miR168, miR397, miR398, miR408 and miR528 tend to increase, regardless of photoperiod absence/presence, demonstrating the great influence of hormone on specific miRNA expression during plant regeneration (Chávez-Hernández et al., 2015). However, photoperiod seems to influence miR164a, miR167a and miR168a expression levels that increase when callus is cultured in the light as well as some miRNAs targets (Chávez-Hernández et al., 2015). The increase of those miRNAs reveals their stage-specific functions in bud formation (Wu et al., 2011; Liu et al., 2014).

## Long non-coding RNAs

lncRNAs are transcripts longer than 200 nt that are transcribed by Pol II and can be grouped in linear and circular lncRNAs (lincRNAs and circRNAs, respectively) and sub-grouped according to the genome region from which they arise, i.e., intergenic, intronic and coding region (Yu et al., 2019). These poorly conserved RNA molecules function as precursors of miRNAs and other sRNAs (Liu et al., 2015) and regulate chromatin remodelling, transcription process and post-transcriptional processing (Mercer et al., 2009).

Compared to humans and other animals, only a few plant lncRNAs have been studied (Zhang et al., 2019b). Despite the limited functional characterization of most lncRNAs, studies so far have uncovered a wide range of possible functions and molecular mechanisms mediated by plant lncRNA activities (Kim and Sung, 2012; Datta and Paul, 2019). Acting in cis or trans modes, lncRNAs regulate the expression of neighbouring or distant genes, respectively, during different plant developmental processes (Yu et al., 2019). Although recent studies have exposed the role of lncRNAs in the control of cell differentiation and pluripotency maintenance in animal stem cells (Guttman et al., 2011; Chen and Zhang, 2016), similar evidence was not yet fully described for plant cells. Nevertheless, an increasing number of functional studies suggest the contributions of lncRNAs as essential modulators in plant responses to abiotic stresses (Urquiaga et al., 2021).

Indeed, a few reports describe the role of lncRNAs during the induction of SE, a stress-induced plant regeneration process. During SE in longan, some lncRNAs were found differentially expressed and revealed to be involved in gene expression regulation during the process by regulatory networks with miRNAs and mRNAs (Chen et al., 2018). For instance, some lncRNAs function as blockers of miRNA cleavage, which greatly affect the regulation of plant cell differentiation and the development process (Chen et al., 2018). This is the case of the lncRNA INDUCED BY PHOSPHATE STARVATION1 (IPS1) that acts as a target-mimic of miR399 and averts the cleavage of its target mRNA (Franco-Zorrilla et al., 2007). Also, in white spruce, the stress-induced SE was reported to be regulated by a lncRNA-miRNA-mRNA network (Gao et al., 2022). In this study, three lncRNAs (MSTRG.33602.1, MSTRG.505746.1, and MSTRG.1070680.1) were found to positively regulate target genes involved in stress response, auxin signal transduction and early somatic embryo development. Having a protective, or sponge effect, for miRNAs targeting these genes, these lncRNAs are therefore involved in SE and ultimately contribute to the embryogenicity of mature somatic embryos (Gao et al., 2022).

As cell reprogramming inherent to plant regeneration can be understood as a stress-responsive event, the increasing number of lncRNAs described with a regulatory role in plant development and response to various stresses can also be understood as being related to plant developmental plasticity. For instance, the lncRNA LONG DAY-SPECIFIC MALE-FERTILITY-ASSOCIATED RNA (LDMAR) was found to be essential for rice fertility (Ding et al., 2012). As a lncRNA involved in pollen development, LDMAR could also be involved in other developmental transitions such as the ones that occur during plant regeneration. Another example is the case of asHSFB2a, which controls the expression of the heat shock factor HSFB2a after heat stress with influence on vegetative and gametophytic development in *Arabidopsis* (Wunderlich et al., 2014). With a validated role in development transitions, more studies in the asHSFB2a action could bring to light its participation in cell reprogramming. BoNR8 (AtR8 lncRNA homolog in *Brassica oleracea*) negatively affects seed germination, seedling root growth and silique development in Arabidopsis (Wu et al., 2019) Localized in the epidermal tissues of the root elongation zone and induced by abiotic stresses and auxins, this lncRNA is a potential regulator of *in vitro*-induced regeneration processes.

The lncRNAs involved in specific developmental transitions are possible candidates to study as cell reprogramming regulators as well. The lncRNA ALTERNATIVE SPLICING COMPETITOR (ASCO) is an alternative splicing competitor in *Arabidopsis*, that modulates the splicing patterns of some genes and introduces changes during specific developmental transitions, such as the ability to form lateral roots from pericycle cells (Bardou et al., 2014). In the same way, the lncRNAs related to flowering, such as COLD-INDUCED LONG ANTISENSE INTERGENIC NON-CODING RNA (COOLAIR) (Chen and Penfield, 2018), may also affect the developmental transitions that occur during pluripotency acquisition and expression.

**Figure 3:**
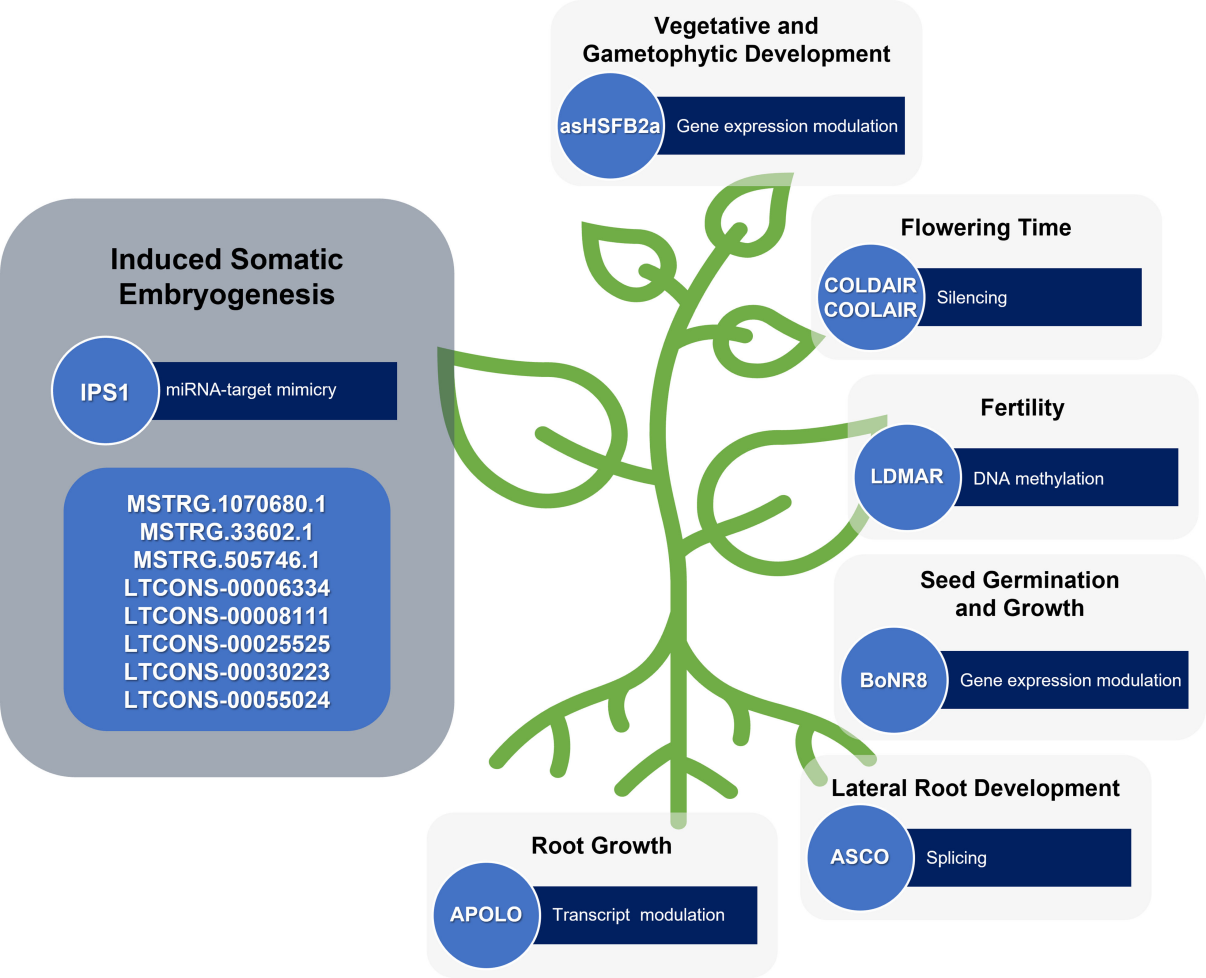
lncRNAs involved in induced plant somatic embryogenesis and other developmental processes. Some lncRNAs still require functional validation.

Since plant regeneration is an auxin-dependent process, the lncRNAs involved in plant development through auxin transport and signalling may also be important players during this process. Accordingly, five lncRNAs (LTCONS-00006334, LTCONS-00008111, LTCONS-00025525, LTCONS-00030223 and LTCONS-00055024) targeting ARFs were found differentially expressed during longan early SE (Chen et al., 2018). Another lncRNA involved in auxin signalling is AUXIN-REGULATED PROMOTER LOOP (APOLO) which is involved in the modulation of PINOID (PID), a key regulator of polar auxin transport during root growth in *Arabidopsis* (Ariel et al., 2014) (**Figure 3**).

## Other ncRNAs

snRNAs are a class of ncRNAs with 100–200 nt in length. These uridine-rich sRNAs constitute the spliceosome, the molecular machinery that catalyzes pre-mRNA splicing (Will and Lührmann, 2011). It was demonstrated that to achieve *in vitro* cell dedifferentiation and organogenesis, higher levels of snRNAs are required than those for seedling development (Ohtani et al., 2015). This suggests the critical role of the splicing capacity in cell pluripotency expression.

siRNAs are double-stranded 20-24 nt long RNA molecules. In plants, they include heterochromatic siRNAs (hc-siRNAs), natural antisense transcript siRNAs (nat-siRNAs), trans-acting siRNAs (tasiRNAs), repeat-associated siRNAs (ra-siRNAs) and long siRNAs (lsiRNAs) (Zhang et al., 2019a). siRNAs lead to gene silencing acting either at the transcriptional level, by inducing epigenetic modifications such as DNA and/or histone methylation, or at the posttranscriptional level, inducing mRNA degradation (Borges and Martienssen, 2015). *In vitro* cultured plant cells undergo extensive epigenetic reprogramming which results in changes in the DNA methylation patterns (Zhang et al., 2018). The increase or decrease of 24 nt siRNAs is correlated with hyper or hypomethylation of the corresponding target genes, respectively (Matzke et al., 2009). Thus, several studies have pointed out a relation between the amount of 24 nt siRNAs and the tissue embryogenic potential, by their impact on genetic reprogramming (Elhiti et al., 2013). In maize, a decrease in the 24 nt siRNAs population is associated with the establishment of embryogenic callus (Alejandri-Ramírez et al., 2018) and, in turn, an increase was observed in fewer embryogenic tissues (Juárez-González et al., 2019). On the contrary, in citrus, a lower abundance of 24 nt siRNAs in non-embryogenic callus has been reported (Wu et al., 2015). Likewise, in grapevine, larger amounts of 24 nt siRNAs were found in embryogenic samples compared to non-embryogenic (Dal Santo et al., 2022). Also, an overrepresentation of 24 nt siRNAs was associated with SE synchronism (Zhang et al., 2014). Thus, as noted by Dal Santo et al. (2022), a readjustment of 24 nt siRNAs appears to be essential for embryogenic commitment.

The multigenerational protection against invasive transposable elements has been described as an important function of 24 nt ncRNAs, particularly at late seed set in conifers, altering developmental programs (Liu and El-Kassaby, 2017).

Recently, López-Ruiz et al. (2020) revealed a dynamic tasiRNAs regulation mediated by ARFs in *in vitro* plant regeneration. The authors concluded that miR390 and tasiRNAs-ARFs contribute differently to the embryogenic potential depending on the plant species and the *in vitro* culture process (López-Ruiz et al., 2020). For instance, in cotton embryogenic callus, three tasiRNAs (TAS3a, TAS3b, and TAS3d) were found up-regulated while TAS3c was down-regulated (Yang et al., 2013), pointing to a role of these RNAs in SE regulation.

Recently, chromatin-enriched ncRNAs (cheRNAs) were identified as positive regulators of genes related to somatic cell reprogramming, being required for cell dedifferentiation and plant regeneration ability in rice (Zhang et al., 2022).

Despite not being essential for the restart of cell division during tissue culture, rRNA transcription is upregulated and required during the dedifferentiation of plant cells (Ohtani, 2015).

## Conclusions

As important genetic and epigenetic regulators, ncRNAs have proven to be essential to achieve pluripotency, and hence, plant regeneration capacity, through gene expression regulation and chromatin remodelling. Indeed, stress-enhanced embryogenicity is achieved through ncRNAs networks that modulate the epigenetic state of the somatic cell towards pluripotency. As stated in this review, an important class of ncRNAs, miRNAs, are proven as key elements in plant regeneration and totipotency, and an increasing amount of evidence integrates their action with lncRNAs that target important genes in such developmental mechanisms. Nevertheless, and despite the growing number of works that demonstrate differential expression of lncRNAs in regeneration processes, there are no functional studies on lncRNAs in induced plant cell reprogramming and regeneration.

Once ncRNAs are gene expression modulators, variation of their expression, by overexpression or downregulation, alters the expression of specific key genes of cell reprogramming which in turn could improve *in vitro* regeneration processes. For instance, overexpression of embryogenic-related miRNAs, such as miR156, would increase explants’ regeneration capacity. In the same way, downregulation of stress- and nutrient-transport-related miRNAs could unlock embryogenic potential acquisition.

Despite appreciable success in understanding the role of miRNAs in plant development, the functions and biological mechanisms of other emerging ncRNAs are still unclear. Intensive efforts are needed to ascertain the functional and regulatory role of ncRNAs in plant cell reprogramming. This is crucial to modulate and improve *in vitro*-induced regeneration processes.

Studies have demonstrated multiple mechanisms of lncRNAs in animals, such as lncRNA-encoded peptides and interaction with DNA or RNA methylases and demethylases; however, the mechanism of action of lncRNAs to regulate RNA methylation of mRNA and protein expression has not been reported in plants. Although plant lncRNAs involved in these functions have not been reported yet, the increasing amount of evidence from other biological systems gives reasons to believe that many of the unknown functions of lncRNAs will be realized through these nodes. The rapid evolution in the integration of data generated by different omics tools is allowing better correlations between coding and non-coding transcriptomes, particularly in the interaction between mRNA-miRNA-lncRNA, revealing new functional interactions whose validation will be crucial for a full understanding of the regulatory mechanisms and their possible modulation. These mechanisms of ncRNA action in plants can provide directions for future research, and more functions of plant ncRNAs can be determined.

## Author contributions

DC and SC conceived the idea of the review and prepared the initial outline. DC wrote the first draft. JC and SC were involved in manuscript refinement. All authors contributed to the article and approved the submitted version.

## Funding

This work was supported by the Foundation for Science and Technology (Portugal) through a doctoral research fellowship (SFRH/BD/136925/2018) awarded to DC and was carried out at the R&D Unit Centre for Functional Ecology - Science for People and the Planet (CFE), with reference UIDB/04004/2020, financed by FCT/MCTES through national funds (PIDDAC).

## Conflict of interest

The authors declare that the research was conducted in the absence of any commercial or financial relationships that could be construed as a potential conflict of interest.

## Publisher’s note

All claims expressed in this article are solely those of the authors and do not necessarily represent those of their affiliated organizations, or those of the publisher, the editors and the reviewers. Any product that may be evaluated in this article, or claim that may be made by its manufacturer, is not guaranteed or endorsed by the publisher.
